# Supplementary material for: Blockade of Human α7 Nicotinic Acetylcholine Receptor by α-Conotoxin ImI Dendrimer: Insight from Computational Simulations
Source: Mar Drugs. 2019 May 23;17(5):303. doi: 10.3390/md17050303 (PMC6563025; doi:10.3390/md17050303)
Supplement: Supplementary file 1 [file marinedrugs-17-00303-s001.pdf]

Blockade of Human α7 Nicotinic Acetylcholine Receptor by α-Conotoxin ImI Dendrimer: Insight from Computational Simulations

Supplementary Materials

Xiaoxiao Xu[1,2], Jiazhen Liang[1,2], Zheyu Zhang[1], Tao Jiang[1,2] and Rilei Yu[1,2,3*]

[1] Key Laboratory of Marine Drugs, Chinese Ministry of Education, School of Medicine and Pharmacy, Ocean University of China, Qingdao 266003, China; 21170831073@stu.ouc.edu.cn (X.X.); 11180822010@stu.ouc.edu.cn (J.L.); zzy8617@stu.ouc.edu.cn (Z.Z.); jiangtao@ouc.edu.cn (T.J.)

[2] Laboratory for Marine Drugs and Bioproducts of Qingdao National Laboratory for Marine Science and Technology, Qingdao 266003, China

[3] Innovation Center for Marine Drug Screening & Evaluation, Qingdao National Laboratory for Marine Science and Technology, Qingdao 266003, China

* Correspondence: ryu@ouc.edu.cn; Tel.: +86-138-6986-2306

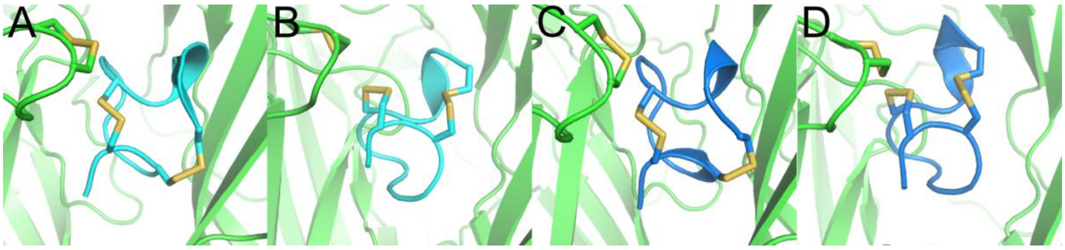

Figure S1. Binding mode of α-ImI monomer and dimer. (A, B) represent the binding mode of α-ImI of the dimer (light blue) with hα7 nAChR, and (C, D) represent the binding mode of α-ImI monomer (deep blue) with the same binding sites of hα7 nAChR.

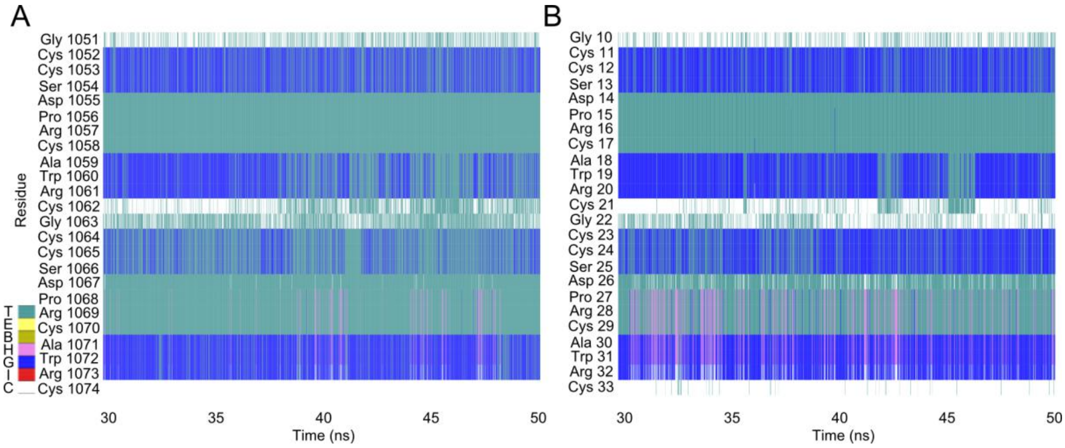

Figure S2. Characterization chart of secondary structure of each α-ImI in 2×ImI-dendrimer. A represents the secondary structure diagram of two α-ImI monomers. B represents the secondary structure diagram of two α-ImI in 2×ImI-dendrimer. Color key for secondary structure plots are as follows: T (Turn); E (Extended configuration); B (Isolated bridge); H (Alpha helix); G (3-10 Helix); I (Pi-helix); C (coil).

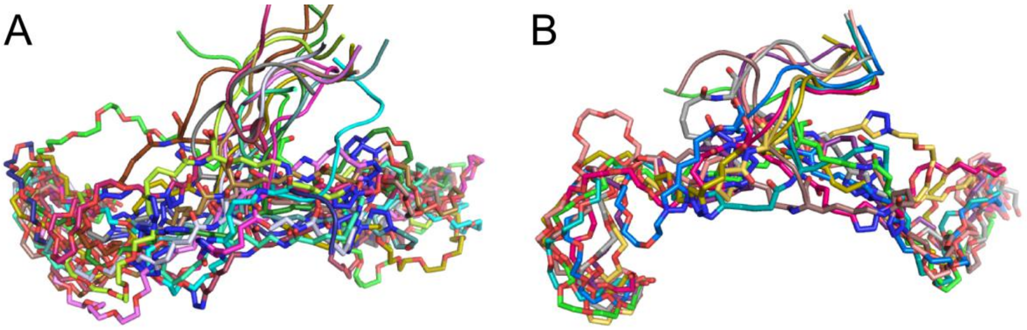

Figure S3. Conformational superposition diagram of the linker. (A) Frames extracted in the former 14 ns with even time intervals and the last frame (green) at 50ns. (B) The averagely extracted frames from 14th ns to 34th ns and the last frame (green) in 50ns.

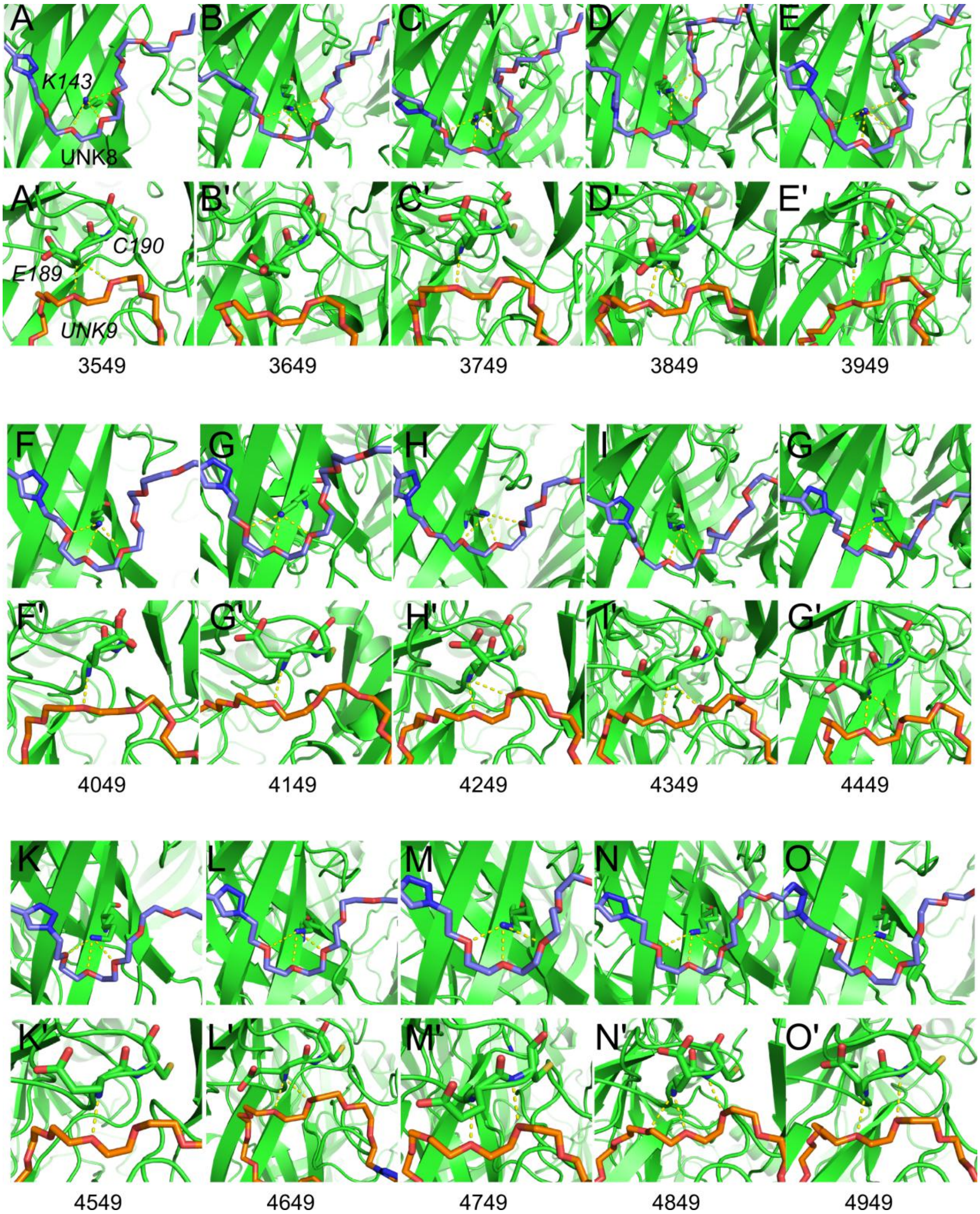

Figure S4. Cluster analysis diagram of the linker between 34 ns to 50 ns. 3549 - 4949 represent the extracted conformation number. The hα7 nAChR was shown in green and two parts of the linker was colored in orange (UNK9) and purple (UNK8), respectively.

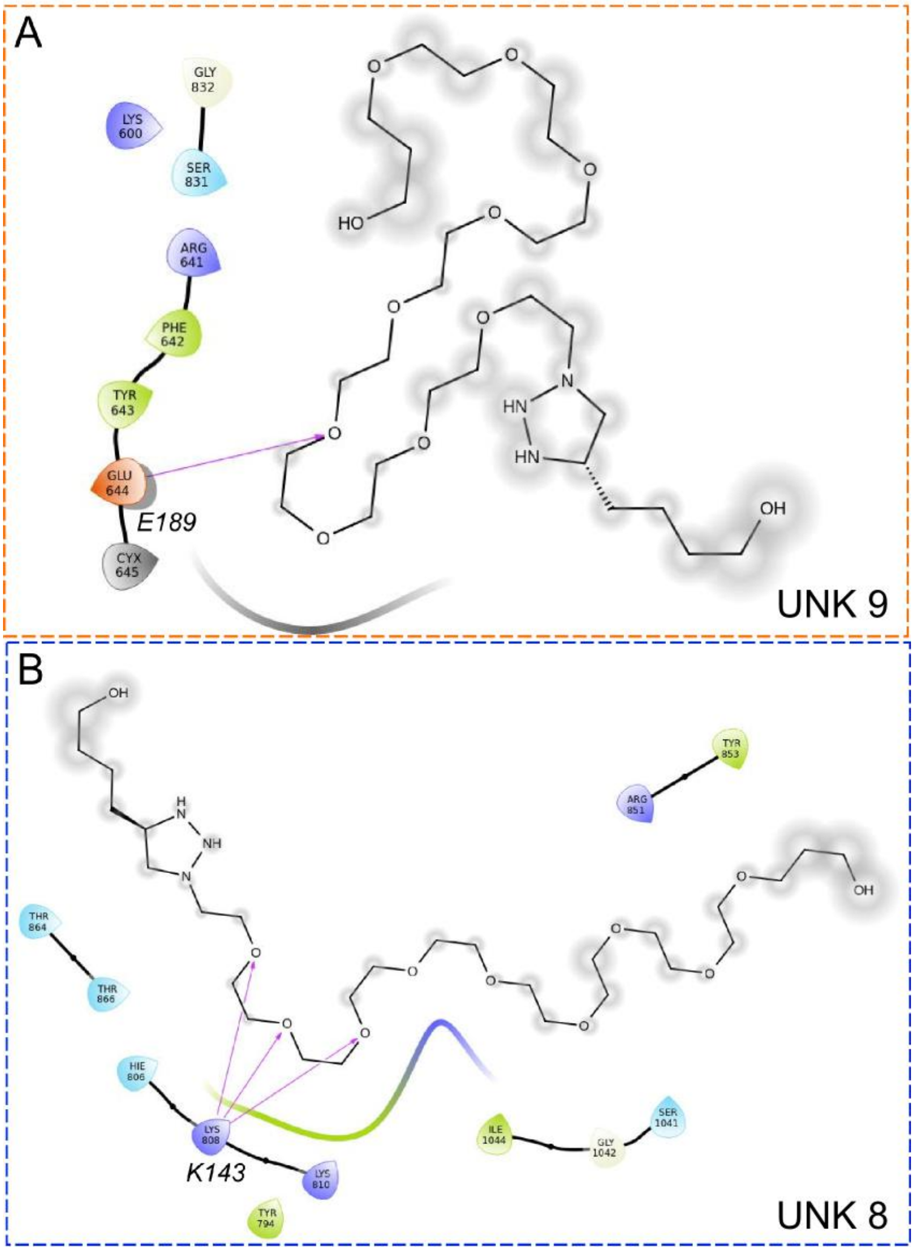

Figure S5. 2D interaction diagrams of the linker. (A) Binding mode of UNK9/hα7-nAChR; (B) Binding mode of UNK8/hα7-nAChR.

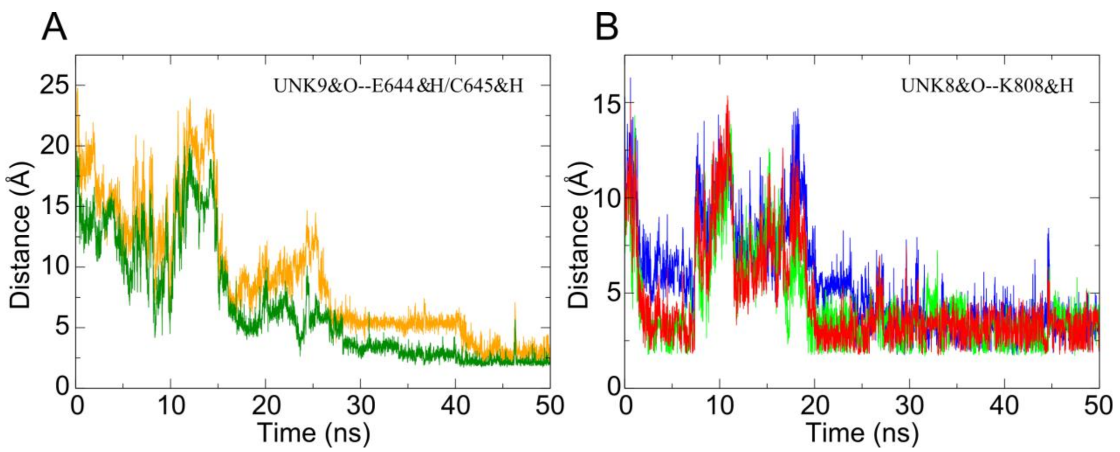

Figure S6. H-bond analysis of UNK with hα7 nAChR. (A) Evolution of the distance between O atom of UNK9 and H atom of E644/C645 in hα7 nAChR. (B) Evolution of the distance between O atom of UNK8 and H atom of K808 in hα7 nAChR.

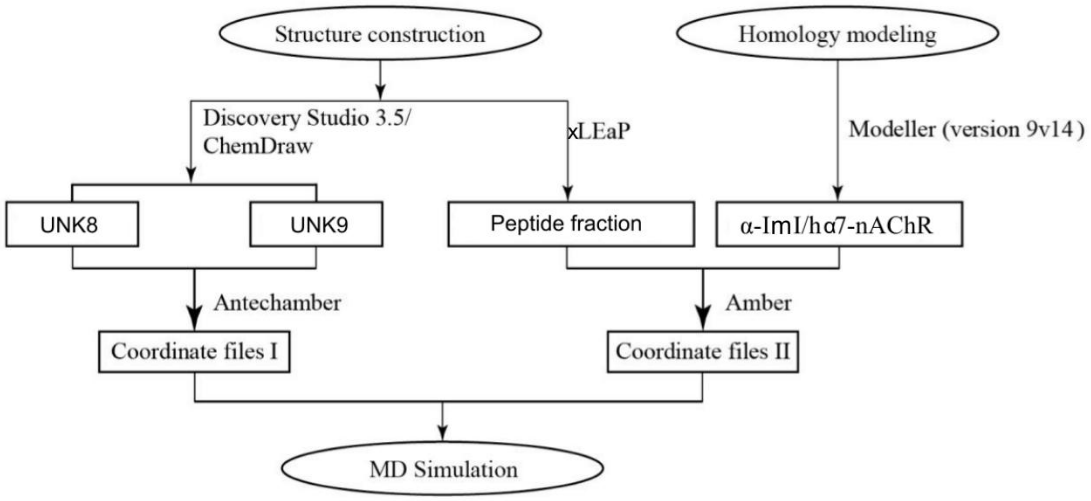

Figure S7. Flowchart for building the α-ImI dendrimer model. The UNK8 and UNK9 represent the PEG spacer unit. The peptide fragment consists of 7 amino acid residues (Lys-Gly-Arg-Arg-Arg-Arg-Gly).

Table S1. Assigned partial charges for the UNK8/UNK9

UNK8/UNK9.prepin
0 0 2<br><br>This is a remark line<br>molecule.res<br>UNK INT 0<br>CORRECT OMIT DU BEG<br>0.0000

1	DUMM	DU	M	0	-1	-2	0.000	.0	.0	.00000
2	DUMM	DU	M	1	0	-1	1.449	.0	.0	.00000
3	DUMM	DU	M	2	1	0	1.523	111.21	.0	.00000
4	C6	C	M	3	2	1	1.540	111.208	-180.000	0.564600
5	O1	O	E	4	3	2	1.209	119.705	7.057	-0.527380
6	C5	CT	M	4	3	2	1.519	117.177	-173.884	-0.199506
7	H9	HC	E	6	4	3	1.116	107.629	57.858	0.069663
8	H10	HC	E	6	4	3	1.116	107.529	-58.215	0.069663
9	C4	CT	M	6	4	3	1.537	114.565	179.857	-0.077441
10	H7	HC	E	9	6	4	1.117	109.340	58.080	0.055171
11	H8	HC	E	9	6	4	1.117	109.386	-59.553	0.055171
12	C	CT	M	9	6	4	1.537	111.992	179.446	-0.094750
13	H3	HC	E	12	9	6	1.114	109.780	56.844	0.060668
14	H4	HC	E	12	9	6	1.115	110.073	-61.118	0.060668
15	C8	CV	M	12	9	6	1.502	111.272	178.737	0.260462
16	N	NB	S	15	12	9	1.267	122.016	-115.407	-0.355188
17	N1	NB	E	16	15	12	1.257	104.933	179.323	-0.165888
18	C3	CA	M	15	12	9	1.338	127.535	63.866	-0.222718
19	H2	H4	E	18	15	12	1.093	131.493	0.242	0.194897
20	N2	N*	M	18	15	12	1.266	102.399	-179.302	0.043377
21	C1	CT	M	20	18	15	1.473	120.162	179.076	0.049274
22	H5	H1	E	21	20	18	1.116	110.956	30.268	0.079158
23	H6	H1	E	21	20	18	1.115	109.116	147.344	0.079158
24	C7	CT	M	21	20	18	1.529	110.132	-91.817	0.113340
25	H11	H1	E	24	21	20	1.114	109.960	-61.576	0.046675
26	H12	H1	E	24	21	20	1.114	109.510	58.710	0.046675
27	O	OS	M	24	21	20	1.407	109.017	178.315	-0.429828
28	C2	CT	M	27	24	21	1.407	112.341	-178.986	0.128332
29	H13	H1	E	28	27	24	1.114	109.526	-61.485	0.047675
30	H14	H1	E	28	27	24	1.116	109.333	58.349	0.047675
31	C9	CT	M	28	27	24	1.522	109.014	178.067	0.128332
32	H15	H1	E	31	28	27	1.114	109.693	-57.608	0.046176
33	H16	H1	E	31	28	27	1.115	110.309	63.073	0.046176
34	O2	OS	M	31	28	27	1.406	108.370	-177.015	-0.429828
35	C10	CT	M	34	31	28	1.406	112.860	177.797	0.128332
36	H17	H1	E	35	34	31	1.115	109.407	-58.049	0.041678
37	H18	H1	E	35	34	31	1.115	109.565	62.036	0.041678
38	C11	CT	M	35	34	31	1.522	108.309	-177.522	0.123335
39	H19	H1	E	38	35	34	1.114	109.938	-63.670	0.041178
40	H20	H1	E	38	35	34	1.113	109.162	56.107	0.041178
41	O3	OS	M	38	35	34	1.407	109.029	176.390	-0.421824
42	C12	CT	M	41	38	35	1.409	113.310	-174.532	0.129331
43	H21	H1	E	42	41	38	1.116	106.727	-158.010	0.062667
44	H22	H1	E	42	41	38	1.113	110.409	-41.714	0.062667

45	C13	CT	M	42	41	38	1.523	111.371	83.423	0.091352
46	H23	H1	E	45	42	41	1.110	110.550	-61.618	0.035681
47	H24	H1	E	45	42	41	1.115	109.777	58.755	0.035681
48	O4	OS	M	45	42	41	1.408	108.795	178.286	-0.417821
49	C14	CT	M	48	45	42	1.409	113.469	177.905	0.128332
50	H25	H1	E	49	48	45	1.117	106.522	160.760	0.062667
51	H26	H1	E	49	48	45	1.112	110.385	44.714	0.062667
52	C15	CT	M	49	48	45	1.523	111.807	-80.724	0.097348
53	H27	H1	E	52	49	48	1.113	110.886	64.780	0.034682
54	H28	H1	E	52	49	48	1.115	109.961	-56.210	0.034682
55	O5	OS	M	52	49	48	1.408	108.687	-175.881	-0.425826
56	C16	CT	M	55	52	49	1.408	112.424	-179.241	0.127332
57	H29	H1	E	56	55	52	1.115	109.740	-60.479	0.045176
58	H30	H1	E	56	55	52	1.115	109.127	58.977	0.045176
59	C17	CT	M	56	55	52	1.521	109.403	178.355	0.128332
60	H31	H1	E	59	56	55	1.115	109.310	-172.858	0.045176
61	H32	H1	E	59	56	55	1.115	110.178	-52.928	0.045176
62	O6	OS	M	59	56	55	1.408	109.360	67.856	-0.429828
63	C18	CT	M	62	59	56	1.407	112.347	179.391	0.128332
64	H33	H1	E	63	62	59	1.114	109.460	-58.834	0.041178
65	H34	H1	E	63	62	59	1.116	109.424	60.994	0.041178
66	C19	CT	M	63	62	59	1.521	108.906	-178.794	0.130331
67	H35	H1	E	66	63	62	1.114	110.080	-61.267	0.042178
68	H36	H1	E	66	63	62	1.116	109.801	59.293	0.042178
69	O7	OS	M	66	63	62	1.407	108.554	178.873	-0.430828
70	C20	CT	M	69	66	63	1.406	112.682	-178.883	0.130331
71	H37	H1	E	70	69	66	1.115	109.522	-60.139	0.042677
72	H38	H1	E	70	69	66	1.115	109.449	59.807	0.042677
73	C21	CT	M	70	69	66	1.521	108.590	179.754	0.128332
74	H39	H1	E	73	70	69	1.115	109.866	-59.106	0.040678
75	H40	H1	E	73	70	69	1.115	109.927	61.329	0.040678
76	O8	OS	M	73	70	69	1.407	108.812	-178.791	-0.428827
77	C22	CT	M	76	73	70	1.407	112.520	-179.830	0.128332
78	H41	H1	E	77	76	73	1.115	109.172	-59.713	0.044177
79	H42	H1	E	77	76	73	1.114	109.766	59.951	0.044177
80	C23	CT	M	77	76	73	1.522	109.174	-179.279	0.128332
81	H43	H1	E	80	77	76	1.115	109.544	174.781	0.045676
82	H44	H1	E	80	77	76	1.115	110.076	54.732	0.045676
83	O9	OS	M	80	77	76	1.407	109.192	-65.791	-0.432829
84	C24	CT	M	83	80	77	1.407	112.586	-179.879	0.138327
85	H45	H1	E	84	83	80	1.114	109.283	-59.380	0.040678
86	H46	H1	E	84	83	80	1.115	109.216	60.602	0.040678
87	C25	CT	M	84	83	80	1.529	108.548	-179.338	-0.206509
88	H47	HC	E	87	84	83	1.116	109.500	-58.956	0.082656

```
  89   H48    HC      E    87   84   83       1.116     109.479      59.198   0.082656
  90   C26    C       M    87   84   83       1.519     114.477   -179.881   0.566599
  91   O10    O       E    90   87   84       1.210     122.796       0.576 -0.526379


LOOP
   N2    N1

IMPROPER
   -M    C5    C6    O1
   C3     C    C8     N
   C8    H2    C3    N2
   C3    C1    N2    N1
   +M   C25   C26   O10

DONE
STOP
```
